# Day–night gene expression reveals circadian gene *disco* as a candidate for diel-niche evolution in moths

**DOI:** 10.1098/rspb.2024.0591

**Published:** 2024-08-28

**Authors:** Yash Sondhi, Rebeccah L. Messcher, Anthony J. Bellantuono, Caroline G. Storer, Scott D. Cinel, R. Keating Godfrey, Andrew J. Mongue, Yi-Ming Weng, Deborah Glass, Ryan A. St Laurent, Chris A. Hamilton, Chandra Earl, Colin J. Brislawn, Ian J. Kitching, Seth M. Bybee, Jamie C. Theobald, Akito Y. Kawahara

**Affiliations:** ^1^ McGuire Center for Lepidoptera and Biodiversity, Florida Museum of Natural History, University of Florida, Gainesville, FL 32611, USA; ^2^ Department of Biology, Florida International University, Miami, FL 33174, USA; ^3^ Institute for Environment, Florida International University, Miami, FL 33174, USA; ^4^ Department of Entomology and Nematology, University of Florida, Gainesville, FL 32611, USA; ^5^ School of Life Sciences, University of Sussex, Sussex House, Brighton BN1 9RH, UK; ^6^ Natural History Museum, Cromwell Road, London SW7 5BD, UK; ^7^ Department of Entomology, Smithsonian Institution, National Museum of Natural History, Washington, DC, USA; ^8^ Department of Entomology, Plant Pathology & Nematology, University of Idaho, Moscow, ID 83844, USA; ^9^ Biodiversity Knowledge Integration Center, School of Life Sciences, Arizona State University, Tempe, AZ 852281, USA; ^10^ Independent Researcher, Huntingdon, Pennsylvania 16652, USA; ^11^ Department of Biology, Monte L. Bean Museum, Brigham Young University, 4102 Life Science Building, Provo, UT 84602, USA

**Keywords:** chronobiology, de novo transcriptome, Lepidoptera, light cycle, RNA-Seq, temporal

## Abstract

Temporal ecological niche partitioning is an underappreciated driver of speciation. While insects have long been models for circadian biology, the genes and circuits that allow adaptive changes in diel-niches remain poorly understood. We compared gene expression in closely related day- and night-active non-model wild silk moths, with otherwise similar ecologies. Using an ortholog-based pipeline to compare RNA-Seq patterns across two moth species, we find over 25 pairs of gene orthologs showing differential expression. Notably, the gene *disco,* involved in circadian control, optic lobe and clock neuron development in *Drosophila*, shows robust adult circadian mRNA cycling in moth heads. *Disco* is highly conserved in moths and has additional zinc-finger domains with specific nocturnal and diurnal mutations. We propose *disco* as a candidate gene for the diversification of temporal diel-niche in moths.

## 1. Introduction

Ecological niche, including the temporal partitioning of activities within a day (diel-niche), is an underappreciated driver of speciation and adaptive evolution [1]. Partitioning can help organisms exploit resources, avoid competition and reduce predation risk [2–7]. Differences in physiology and performance at various temperatures, light levels and humidity can also drive partitioning [8]. In insects, circadian rhythms are crucial for regulating daily locomotor, reproductive behaviour and feeding activity [5,9–11]. Over the past few decades, research in *Drosophila* has led to a better understanding of the molecular and neural mechanisms underlying insect circadian rhythms [12,13]. They are governed by transcriptional–translational feedback loops, self-sustained molecular oscillations that affect patterns of gene expression at the cellular level and, due to expression in the central nervous system, scale up to determine whole-organism patterns of activity (reviewed in *D. melanogaster* in [12,14]).

Four key elements have been identified in the circadian control system that may be relevant to temporal niche evolution: (i) genes and proteins that govern the central clock, (ii) neural circuits connecting the clock to downstream processes, (iii) neuropeptides, such as pigment-dispersing factor (PDF), released by these clock neurons to modulate the synchronization within the clock network, and (iv) light detection by photoreceptors and photosensitive molecules, which entrain the clock to match external light cycles. Behaviours with strong diel periodicity that also impact fitness could drive selection on genes and circuits of the molecular clock or on those involved in entrainment to external stimuli.

While the identity and function of core clock genes have been explored extensively in *D. melanogaster* [15–17], the clock proteins period (Per), timeless (Tim) and cryptochrome (Cry) are highly conserved across animals [18,19]. They participate in auto-regulatory feedback loops to produce changes in gene expression on an approximately 24 h cycle. Nearly one-third of cell types in *D. melanogaster* express clock genes [20], but daily circadian patterns only require expression of core molecular clock components in about 150 clock neurons [21–24]. These cluster into 3 dorsal groups (DNs) and 4 lateral groups (LNs) based on cell body location and cluster into 17 groups based on gene expression [13,25]. A subset of LNs and DNs release PDFs and other neuromodulators to keep clock cells synchronized or signal to other neuroendocrine centres [26]. These oscillators can be light entrained via light-sensitive proteins expressed in clock cells (Cry) and peripheral photoreceptors (rhodopsin Rh6) [27,28]. Flies with different activity periods have displayed species-specific expression patterns of Cry and PDF [29].

Comparisons among circadian genes and circuits across evolutionary time scales indicate a high degree of conservation between invertebrates [30–32] and vertebrates [33], and insects serve as useful genetic models of human circadian disorders [32,34,35]. However, since diel switches can occur over shorter evolutionary time scales [29], examining more divergent circadian genes can shed light on circadian evolution. Lepidoptera (the moths and butterflies) contain many extreme diel-niche switches, often between closely related species, and their evolutionary history is well known, making them ideal for studying diel-niche evolution [36,37]. They also have some of the best characterized circadian and sensory genes and circuits, outside of *Drosophila* [38–44]. For example, the wild silk moth family, Saturniidae is an important model for understanding chronobiology [45,46]. Moths are thus a useful system to explore the adaptive significance of circadian rhythms in the context of ecological niche partitioning [47,48].

We investigate two closely related saturniid moth species, the pink-striped oakworm moth (*Anisota pellucida*) and the rosy maple moth (*Dryocampa rubicunda*) that overlap in geographic distribution and habitat but differ in daily activity patterns, with *Anisota* being diurnal and *Dryocampa* being nocturnal. These species diverged approximately 3.8 Ma [49] and represent a relatively recent shift in diel-niche. We compare the day–night cycling genes to identify candidate drivers of temporal partitioning. We identify differentially expressed orthologs and focus our analysis on gene pairs that show day–night cycling patterns in both species. We consider genes that switch from upregulation to downregulation or vice versa across species, and those that are strongly differentially regulated in only one species, as suitable candidates for being involved in diel shifts. We explore the evolution of the gene *disco*, one of these candidates, with known circadian function in flies. We use modelling to predict and compare its protein structure, function and evolutionary conservation in moths and flies.

## 2. Methods

### (a) Insect rearing and sampling design

Moths were reared under natural light–dark cycles at room temperature (25°C). In total, three–five adult males were sampled 2 days after eclosion at the two time points, midday (‘day’) and midnight (electronic supplementary material S1). Moths were decapitated and heads flash-frozen using liquid nitrogen and stored at −80°C.

### (b) RNA extraction, library preparation and sequencing

Tissues were homogenized using a bead beater and extracted using a modified Trizol extraction protocol (electronic supplementary material, data15). RNA clean-up was done using a Thermo Scientific RapidOut DNA Removal Kit (Waltham, MA, USA). Samples were shipped overnight to the NERC-NBAF, Liverpool, UK after dehydrating in a biosafety chamber using GenTegraRNA tubes from (GenTegra, CA, USA) and assessed for DNA and RNA yields. We selected samples for library preparation based on RNA quality to ensure equal sampling across treatments. RNA libraries were prepared using the NEB polyA selection and NEBNext Ultra II directional stranded kit (New England Biolabs, MA, USA) suitable for low input yields. Twelve samples were run on one lane of an Illumina NovaSeq using SP chemistry (Paired-end, 2 × 150 bp sequencing).

### (c) Read trimming and clean-up

For *A. pellucida* and *D. rubicunda*, read trimming was undertaken by the NERC-NBAF core, and the raw Fastq files were trimmed for the presence of Illumina adapter sequences using Cutadapt v. 1.2 [50]. The option ‘-O 3’ was used, so that the 3′ end of any read that matched the adapter sequence for greater than 3 bp was trimmed. Reads were further trimmed using Sickle v. 1.200 [51] with a minimum window quality score of 20. Reads less than 15 bp after trimming were removed.

### (d) De novo transcriptomes, transcriptome library sizes, quality control and annotation

Quality control (QC) was conducted on trimmed reads; library size varied for each species (electronic supplementary material, figure S2). We examined expression data and removed genes with transcripts per million (TPM) less than 1. We combined reads from multiple samples and generated several reference de novo transcriptomes of these assemblers, combined them and measured duplication, completeness and redundancy for the different versions (electronic supplementary material, table S2). Further QC and filtering were conducted using BUSCO v. 5.2.0 [52], TransRate v. 1.0.3 [53], QUAST v. 5.02 [54], CD-HIT v. 4.6.8 [55,56] and cascaded clustering with MMseqs2 v. 12 [57]. We used the least duplicated and most complete assemblies; however, we repeated certain analyses with less conservative assemblies (electronic supplementary material, S1). Transcriptomes were annotated with eggNOG-mapper v. 2.1.9 [58] and OrthoFinder v. 2.5.2 [58,59].

### (e) Differential gene expression enrichment, orthology and network analysis

Reads were mapped using Salmon v. 0.14.1 [60] and differential gene expression analysis was conducted using EdgeR v. 3.38.1 [61] and DESeq2 v. 1.36.0 [62]; the former was used to normalize and test for significantly expressed genes, and the latter was used to normalize and to generate principal component analyses (PCAs) and other summary statistics. Genes were assigned into orthogroups using OrthoFinder v. 2.5.2 [59,63], which used predicted genes from *Bombyx mori*, *Antheraea pernyi* and *Antheraea yammai* as templates [64–66]. These orthogroups were used to transfer annotations from *Bombyx* (described later) or to identify pairs of differentially expressed orthologs using orthologous gene pairs that showed significant fold change (FC) in both species pairs. We performed gene enrichment analysis using GO terms with the tools TopGO v. 2.48.0 [67], ReviGo (https://github.com/rajko-horvat) and ShinyGo v. 0.75c [68] (http://bioinformatics.sdstate.edu/goc/). Gene network analysis was undertaken with WGCNA v. 1.71 [69,70].

### (f) Gene functional annotation

We functionally annotated genes by cross-referencing them with the *B. mori* annotations and adding EggNOG annotations. We divided these into functional groups of vision, smell, hearing, circadian, behaviour and brain using GO terms from amigo (http://amigo.geneontology.org/) (electronic supplementary material, table S8).

### (g) Gene mining and *in silico* evolution

We mined genes of interest from moths and insects using well-annotated genomes on Ensembl and NCBI, insectbase [71]. *Bombyx mori* and *A. pernyi* were taken from the source papers [64–66]. Two sets of analyses were conducted: (i) 18 Bombycoidea moths and their relatives and (ii) 38 insect genomes (electronic supplementary material, data 11). We assigned a reference protein sequence for each gene of interest from the *B. mori*-predicted proteome. We used OrthoFinder v. 2.5.2 to identify orthologs [59,63] and filtered orthogroups containing the reference sequence. To ensure a single sequence per species in each orthogroup, custom python scripts filtered the longest and most similar sequences to the reference.

We ran AlphaFold v. 2.1.2 [72] to predict the three-dimensional structures for each reference *B. mori* sequence. For alignments less than 1500 amino acids and with high conservation scores, calculated using Alistat v. 1.12 [73], we modelled conservation using the webserver of Consurf (https://consurf.tau.ac.il/consurf_index.php) [74–78]. Results were displayed using Jmol first glance viewer (http://firstglance.jmol.org/). Outputs for alpha fold runs and consurf are available as electronic supplementary material data. PyMol v. 2.5.5 [79] was used to align the three-dimensional structures, and Aliview was used to compare alignments. We used InterProScan webserver v. 5.66-98.0 [80,81] (https://www.ebi.ac.uk/interpro/about/interproscan/) and NetPhos v. 3.1 [82,83] for predicting the domains and phosphorylated sites.

## 3. Results

**Figure 1.**
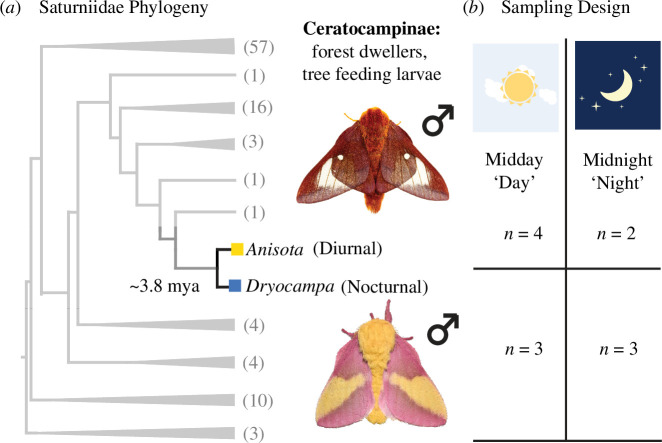
Nocturnal and diurnal moths on a phylogeny with RNA-Seq sampling design. (*a*) Collapsed phylogeny of Saturniidae, adapted to show the placement of the two study species, diurnal *A. pellucida* (pink-striped oakworm moth) and nocturnal *D. rubicunda* (rosy maple moth). Grey numbers on tips represent the number of described genera in the tree before collapsing. Phylogeny adapted from Rougerie *et al*. [49]. (*b*) Sampling design showing the number of replicates sampled for each species and collection period (day/night). Collection of heads was done 2 days post eclosion at midday (sun) and midnight (moon). Photo credits *A. pellucida* © Mike Chapman; *D. rubicunda* (Creative Commons) Andy Reacgo and Chrissy Mclearan.

We compared gene expression across two wild silk moth species, *A. pellucida* and *D. rubicunda* (referred to as *Anisota* and *Dryocampa* hereon) whose males are diurnal and nocturnal, respectively (figure 1, electronic supplementary material, table S1). We generated head (eyes and brain) transcriptomes from moths collected and flash frozen at midday and midnight, referred to as ‘day’ and ‘night’ hereafter. Using multiple programs to assemble high-quality de novo assemblies (electronic supplementary material, table S2), we characterized the level of gene (mRNA) expression mapping reads to them.

### (a) Day–night gene expression patterns switch between nocturnal and diurnal species

**Figure 2.**
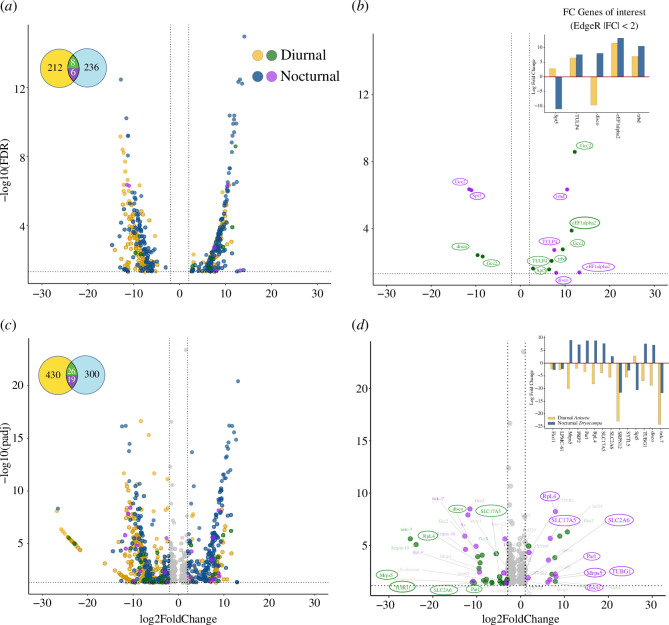
Nocturnal and diurnal species show divergent patterns of gene expression with different analytical approaches (*a,c*): Left panels display volcano plots illustrating fold change and adjusted *p*-values for the significant differentially expressed genes between samples collected at midday and midnight for EdgeR (*a*) and DESeq2 (*c*). Circles at the upper left corner of (*a*) and (*c*) represent the number of genes expressed in both species and colours correspond to FC values for those genes in volcano plots. Yellow and blue represent genes expressed only in the nocturnal or diurnal species, green/purple indicates DEGs present in both species. (*b,d*) Right panels display only genes expressed in both species for EdgeR (*b*) and DESeq2 (*d*). Genes that display opposite trends in expression are highlighted and displayed in the boxplot inset in the upper left corner. Positive fold change indicates night overexpression and negative fold change indicates day overexpression. Genes had false discovery rate (FDR) or adjusted *p*‐value < 0.05 and −2 < FC < 2. Gene names and annotations are from *B. mori*.

We found 350 and 393 significantly differentially expressed genes (DEGs) when comparing day and night treatments for each species (figure 2*a*; electronic supplementary material, table S3,data 1). *Anisota* had more day-upregulated genes (56%), and *Dryocampa* was slightly more night-upregulated (53%). To compare DEG sets between species, we mapped our DEGs to *B. mori* (electronic supplementary material, data S1). Approximately 60% of DEGs from each species had identifiable orthologs in *B. mori* (electronic supplementary material, table S3), and only a small number of DEGs (6–8 genes) overlapped between both species (figure 2*b*). We also replicated this analysis using DESeq2, another differential gene expression (DGE) tool, to ensure that our results were robust to different normalization methods [84]. With DESeq2, we found 498 and 697 DEGs (figure 2*c*; electronic supplementary material, table S3, data S2), with similar *B. mori* annotation rates (61%), although the proportion of day upregulated genes increased considerably in *Anisota* (79%) compared with being more evenly split in *Dryocampa* (50%; electronic supplementary material, table S3). The total number of overlapping genes increased (19–26; figure 2*d*) when using DESeq2. A comparison of the two methods revealed that 174 and 216 genes were shared between *Anisota* and *Dryocampa*, respectively.

### (b) Divergently expressed genes are linked to brain optic lobe, antennal and neural development

To identify important regulators involved in diel-niche evolution, we applied two filtering criteria to our gene expression data. First, we selected genes that exhibited highly significant differential expression in both species. Second, we focused on genes that displayed upregulation or downregulation patterns consistent with the natural diel activity of each species. Our rationale was that this subset of genes was more likely to contain key candidate regulatory genes. To compare DGE overlap between the two species, we grouped transcripts to their matching orthologs from *B. mori*; if two transcripts from different species mapped to the same ortholog, we treated them as being the same. This allowed us to examine overlapping genes between the species to see if any genes switched fold-change sign from positive to negative or vice versa (figure 2, electronic supplementary material, data S3). We found 51 overlapping DEG transcripts that mapped to 28 unique *B. mori* genes. Nine genes showed flipped patterns of expression between the two species, and eight coincided with known diel activity patterns (electronic supplementary material, table S7). Examining gene ontology (GO) annotations and comparing orthologs from flybase (https://flybase.org/), we found genes linked to optic lobe and antennal development (*disco*), locomotion and energy use (*SLC2A6* and *SLC17A5*), brain and neural development (*TUBG1*) and other essential biological processes like transcription, ribosomal translation, protein processing, mitochondrial maintenance (*RpS4, PARL* and *Mrps5*) and wound response (*PRP2*) (figure 2*b,d*; electronic supplementary material, table S7). Of these, only three, *disco, Spt5* and *Gcc2,* were recovered using both methods.

### (c) Gene network analysis identifies diel activity and species-specific co-expressed clusters

**Figure 3.**
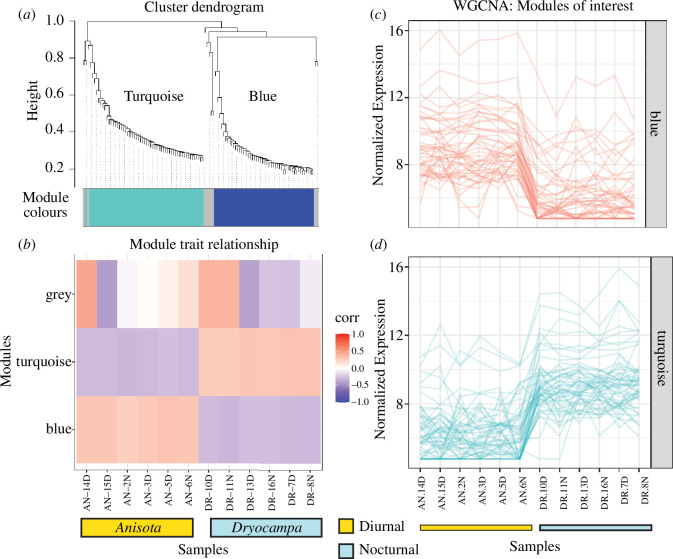
Modules of clustered co-expressed genes grouped using normalized expression. (*a*) Dendrogram showing WGCNA clusters. (*b*) Patterns of gene expression correlate across samples and modules. (*c*) Normalized expression for all genes across samples. Two sets of 50 genes (blue) and (turquoise) have clear species-specific expression patterns. Normalization was done with DESeq2 and reads were mapped to the more stringently filtered transcriptome. A soft power analysis was conducted and a power of 9 was used for the WGCNA analysis. AN: *Anisota*, DR: *Dryocampa*. D and N represent different samples collected at day and night time points.

Identifying highly expressed genes helps understand which genes are activated during certain biological processes. However, determining only those that are highly expressed can often overlook genes with important biological functions [85]. We examined co-expressed genes that may be correlated with diel-niche or RNA collection time. We used WGCNA, a weighted correlation network analysis tool to cluster genes together using their normalized counts [70]. Examining co-expression patterns for each species separately, we found one module in each species that clustered with day–night treatment (cluster-grey60 and cluster-tan) (electronic supplementary material, figure S6 and data S4). Since we were interested in species-specific differences, we re-ran analyses and combined counts from *Anisota* and *Dryocampa,* using only normalized counts for genes that had valid *B. mori* annotations for both species resulting in a list of 2000 genes. Among these, we discovered two clusters (cluster-blue and cluster-turquoise) with 50 genes each that exhibited species-specific expression patterns (figure 3).

### (d) Gene ontology enrichment of photoperiodism, circadian control, muscle and neural growth genes

We used a gene enrichment analysis to determine if GO terms were significantly over-represented in the DEG and WGCNA sets compared with the appropriate background of GO terms. Using TopGo, which allows custom gene sets, we found an over-representation of genes involved in several biological functions (electronic supplementary material, data S5). Neural retinal development, such as folic acid serine, glycine and retinoic acid metabolism had potential links to vision (electronic supplementary material, figure S15). We also used ShinyGo to examine WGCNA clusters (electronic supplementary material, data S5). The less duplicated, filtered transcriptomes were annotated using *B. mori* orthologs (electronic supplementary material, table S4). We examined the enrichment of both tan and grey60 modules, listing the non-redundant terms using ReviGo (electronic supplementary material, figure S7 and data S5). Gene clusters that co-expressed in the same direction together in the day and night treatments of both species included photoperiodism, circadian control, negative phototaxis and nervous system development. Next, we checked for the enrichment of modules that showed species-specific patterns (blue and turquoise; electronic supplementary material, figure S8 and data S5). These included genes involved in muscle proliferation and nerve growth, neural signalling, glycolysis, oxidative stress response and basic cellular functioning such as protein processing and transcriptional regulation.

### (e) Day upregulation of vision genes in the diurnal moth

Since both EdgeR and DESeq2 analyses use different normalization methods and statistical model assumptions to calculate fold change between groups [84,86], we repeated enrichment analyses by examining genes that appeared in both analyses. For *Anisota,* we tested over-enrichment of a smaller subset (FC ≤ −5) of diurnally highly upregulated genes (figure S15, electronic supplementary material, table S5). We found gene enrichment for visual perception, excretion regulation, negative gravitaxis, synaptic plasticity, along with genes associated with other biological processes, such as RNA interference, endopeptidase activity and endocytosis. A reduction in stringency (FC ≤ −2) did not alter results considerably (electronic supplementary material, figure S4). Night-upregulated genes (FC ≥ 2) included ocellary pigment genes, eye-photoreceptor cell development, snRNA processing, post-embryonic development and neurotransmitter secretion, among a host of other processes that may be required for growth, development and metabolism (*wnt* signalling, tricarboxylic acid cycle and cellular response to insulin, glucose transport; electronic supplementary material, figure S4). We also report upregulation (FC ≤ 0) and downregulation (FC ≤ 0) enrichment analyses (electronic supplementary material, data S5).

### (f) Night upregulation of antennal and olfactory brain regions mushroom development genes

We repeated the same analyses for *Dryocampa* and tested the nocturnally upregulated genes (FC ≥ 5). Our results show upregulation in genes associated with mushroom body development, locomotor rhythm, synaptic growth, energy utilization (sialin transport) and mitochondrial translation (figure S15, electronic supplementary material, table S6). A reduction in stringency (|FC| ≥ 2) showed entrainment of the clock cycle and antennal development genes. Genes associated with innate immune response, DNA repair, cell division, histone acetylation, circadian rhythm and retinoid cycle were upregulated during the day, possibly indicating a period of cellular repair during a time when these moths are inactive (electronic supplementary material, figure S5). We also report upregulation (FC ≤ 0) and downregulation (FC ≤ 0) enrichment analyses (electronic supplementary material, data S5).

### (g) Sensory, circadian, eye development and behavioural genes show up in gene network analyses

We combined results from the DEG (EdgeR and DESeq2) and gene network analyses (WGCNA) to create a cumulative list of 1700 transcripts (electronic supplementary material, data S6). Focusing on genes that were recovered across *Anisota* and *Dryocampa* reduced the set to 274 transcripts (electronic supplementary material, data S7). Because many transcripts had poorly annotated *B. mori* hits*,* we improved annotations using the program eggNOG-mapper [58]. We tested if these genes had GO terms associated with sensory, circadian, brain and neural development or behavioural regulatory genes (electronic supplementary material, table S7 and data S8). We found that several genes in each category had GO terms associated with vision and brain development (electronic supplementary material, figure S13 and data S9).

### (h) Expanded genes of interest set identified using a differentially expressed ortholog approach

Since *B. mori* annotations might miss certain genes, we used OrthoFinder to identify orthologs from the de novo transcriptomes of *Anisota* and *Dryocampa*. We used the differentially expressed transcript list from the DESeq2 analyses for each species to find if any transcript had a corresponding ortholog in the other species with differential expression. Thereby identifying pairs of differentially expressed transcripts and their respective fold change values (electronic supplementary material, figure S14). Many of these shared orthologs from the *B. mori* sets; but they also identified 10 additional orthologs (electronic supplementary material, data S6).

### (i) Predicted functional regions and homology patterns identified for genes of interest

**Figure 4.**
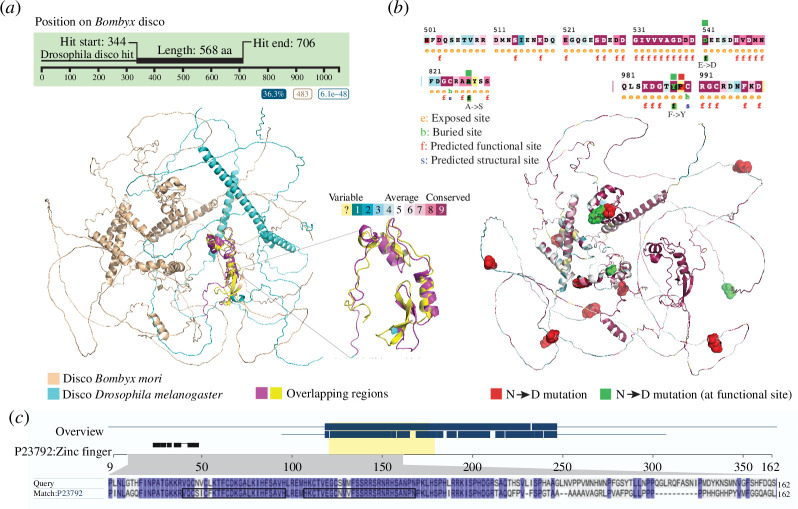
Three-dimensional conservation and domain distribution of *disco* across *Bombyx mori* (1054 aa) and *Drosophila melanogaster* (568 aa). (*a*) Top: *disco* best Uniprot hit in *Drosophila* using default settings (blastp, e-threshold 10, Auto-Blosum62). Bottom: *disco* aligned and superimposed predicted structures for *B. mori* and *D. melanogaster*. Peach colour: *B. mori* alpha-fold predicted structure for *disco,* Cyan: *D. melanogaster* alpha-fold predicted structure for *disco.* (*b*) Top: partial views of the Consurf predicted residues for *disco*. Views encompassing the region where 3/14 mutated sites between nocturnal *Dryocampa* and diurnal *Anisota* overlap with predicted functional residues. Bottom: residues that were mutated between the nocturnal and diurnal species are highlighted on an overlay of the Consurf scores mapped onto the predicted alpha fold structure of *disco* from *B. mori*. Green residues indicate mutated and predicted functional sites, red indicates mutated sites that did not have a predicted residue. (*c*) Overlap of the highly conserved region of *disco*, this region includes the zinc-finger domain that is characteristic of the *disco* transcription factor.

We examined protein and gene evolution for a set of genes which we found were of interest based on results from DGE, WGCNA and GO annotations (figure 4; electronic supplementary material, data S10). We obtained orthologs from publicly available Bombycoidea moth genomes, choosing representative species with the highest identity sequence relative to the *B. mori* reference. We modelled the three-dimensional structure of *B. mori* proteins and mapped the evolutionary conservation onto the three-dimensional predicted structure for proteins above a certain conservation threshold (electronic supplementary material, figure S13 and data S12). These analyses predict structurally and functionally conserved regions of proteins (electronic supplementary material, figure S9 and data 13). We repeated this analysis with 38 insect genomes (electronic supplementary material, data 11) and mapped evolutionary conservation onto three-dimensional protein structure (electronic supplementary material, data S12 and S13). We include results of evolutionary conservation analyses for two regulatory candidates (*disco* and *tk*) that showed varying levels of sequence and protein evolution between insects and moths (electronic supplementary material, figure S10).

### (j) Modelling predicts additional functional zinc finger domains for *disco* in Lepidoptera

The gene *disco* was recovered across multiple analyses. In *D. melanogaster*, *disco* is known for its role in eye development, circadian maintenance, clock neuron development and appendage formation [87–91]. To determine if *disco* was conserved between moths and *D. melanogaster*, we compared the primary sequence and three-dimensional protein structure of *B. mori* and *D. melanogaster*. The sequence length of *disco* in *B. mori* was nearly double that of *D. melanogaster* (figure 4*a*). However, a region spanning over 100 amino acids was highly conserved, contained the zinc-finger domain important for its function and showed strong three-dimensional structural conservation measured by various structural similarity metrics (*whole protein alignment*: RMSD: align = 37.833, super = 2.566, MatchAlign score: align = 540 (2234 atoms), super = 333.1 (554 atoms) versus *alignment of conserved region*: RMSD: align = 2.699, super = 2.566, MatchAlignScore: align = 443 (671 atoms), 351 (615) atoms). These results indicate that the DNA binding domain of *disco* has probably been conserved. However, an additional approximately 500 amino acid region absent in *D. melanogaster* is highly conserved across moths and includes several regions predicted to be functional (figure 4*a*, electronic supplementary material, figure S10). We hypothesize that *disco* has functions in moths that it may not play in flies. To further test this hypothesis, we compared *disco* sequences across *Anisota* and *Dryocampa* finding 23 mutations, 3 of which mapped to the predicted functional region (figure 4*b*, electronic supplementary material, data S14). InterProScan predicted four zinc-finger domains, three in this region, although the CATH-Gene3D databases prediction combined the two separate domains into a single predicted domain (electronic supplementary material, figure S11). We also found 53 sites with predicted phosphorylation potential, with roughly a third of these (18 sites) around the second zinc-finger domain. Reducing the stringency from 0.9 to 0.5 increased the total to 142 sites, which were still enriched around the second domain.

## 4. Discussion

We examined gene expression in the heads of two closely related moths with different activity times: the diurnal *A. pellucida* and the nocturnal *D. rubicunda*. We found 300–700 genes with different expression levels between day and night. Diurnal moths upregulated genes associated with vision while nocturnal moths upregulated genes linked to olfaction and locomotion. Notably, the gene *disconnected (disco*) showed differential expression in both species even accounting for the different models and parameters that were implicated in vision, circadian, hearing, locomotion and brain development in vinegar flies [88,91,92]. Interestingly, *disco* in moths has a larger size and potentially more functional domains compared with its counterpart in fruit flies, suggesting a broader role in moth biology. Several point mutations between *Anisota* and *Dryocampa disco* also mapped to these domains, highlighting the need to further study *disco*.

### (a) Vision and olfaction

Visual systems often accompany diel and photic environment shifts [93], many of these changes are morphological, for example, nocturnal carpenter bees have much larger facets in their eyes than their diurnal counterparts [94], with poorly characterized single genes. Colour vision genes opsin are known to have diel-niche-linked evolution [42,95,96]. However, we found no diel-expression patterns in colour vision opsins, a result corroborated by a recent study [95]. However, we discovered a cerebral opsin (ceropsin), implicated in photoperiodism [97] upregulated during the day.

We also found several eye development genes (*ANKRD17*, *EHD4*, *JAK2* and *TENM2*), phototransduction genes (*PPAP2*, *RDH11*), and retina homeostasis, eye-antennal disc development and photoreceptor cell maintenance genes (*disco, glass*) [98]. Surprisingly a few visual genes, such as *garnet* and *rugose* also appeared to have different isoforms present, showing both day and night upregulation. *Garnet* is an eye-colour mutant gene in flies [99], and *rugose* is implicated in retinal pattern formation [100].

There is evidence for increased investment in olfaction in dim light, with larger mushroom bodies in nocturnal species [93,101,102]. We also found several differently expressed olfactory genes, including those involved in odorant binding (*Obp84a*, *Obp58b*), pheromone response (*tk*), mushroom body development (*DAAM2*, *DST*) and antennal development (*disco*).

### (b) Brain and neural rewiring

We found an upregulation of neural and brain development genes that are also implicated in adult brain plasticity in Lepidoptera and other insects [103–105]. These include genes linked to axon regeneration (*APOD*), central complex development (*ALDH3A2*, *DST*, *OGT*, *Ten-a* and *TENM2*), central nervous system development (*disco*, *RpL4*) and neuropeptide hormonal activity (*tk*). We speculate that plasticity could occur through neural wiring shaping sensory adaptation. Many Lepidoptera show seasonal plasticity in foraging preferences [106], and some override innate preferences for novel visual and olfactory cues after eclosion [107]. Diel-niche and circadian rhythms may also show plasticity, such as *Hyles lineata* showing relatively labile diel-niches possibly driven by temperature and resources [108–110].

### (c) Circadian and behavioural regulators

We found differential expression of genes involved in locomotion (*KCTD15*, *Tk*, *unc-22*) and circadian or rhythmic behaviour (*disco, JAK2*, *OGT* and *SREBF1/SREBF2*) in both species. We also found several key clock genes such as *per* and *tim,* although they were downregulated only in *Dryocampa. Clock-like* (also called *takeout*)*,* another gene under circadian control [111], was expressed in both species, although without any significant upregulation or downregulation. *Clock-like* was moderately conserved in the moths we examined but recovered fewer orthologs across the insects. In *D. melanogaster*, its closest homologs, *Jhbp5* and *takeout*, had only 21–25% sequence identity (electronic supplementary material, data S10 and S14). Despite this, its three-dimensional structure was highly conserved (electronic supplementary material, data S14 and figure S12), indicating likely functional convergence.

### (d) *Disco* as candidate diel-niche gene in adult moths

To identify candidates that might be key regulators of diel-niche, we searched for genes that were (i) expressed in both species, (ii) showed coincident expression patterns with respect to diel-niche, and (iii) played a role in sensory and circadian control. Only *disconnected* (*disco*) fit all criteria.

*Disco* was first described as a locus required for proper optic lobe formation in *Drosophila melanogaster* [112] but is implicated in the disruption of circadian rhythms [87,88,113], potentially through its role in the development of neurosecretory cells in the fly brain [114]. Wild-type flies show a bimodal, crepuscular activity pattern entrained to the external light–dark cycle, which becomes free-running in the absence of the entrainment cue (such as in constant darkness). *Disco* mutants show diurnal entrainment to a light–dark cycle but become arrhythmic in constant darkness [88,114], which appears to be owing to the loss of a set of PDF neurons, from *disco* mutant brains [115,116]. *Pdf* mutants show reduced morning activity bouts in light–dark conditions and, similar to *disco* mutants, become arrhythmic in constant darkness [117,118]. Importantly, these clusters of PDF-expressing cells appear conserved across many insect groups, having been identified in locusts, crickets, stick insects and cockroaches [119,120]. There are no previous studies on *disco* specifically in moths, but publicly available bulk RNA-Seq data from *B. mori* and *Manduca sexta* show *disco* expression in adult in heads and antennae with high larval but minimal pupal expression [65,121]. However, single cell-expression analyses, antibody staining and mRNA *in situ* hybridization are needed to determine if *disco* is expressed in or required for the development of clock neurons in Lepidoptera [122].

The *disco* gene encodes the acid zinc-finger transcription factor, 500 amino acids in length in *D. melanogaster* and 1000 in *B. mori*. Our modelling showed that an approximately 150 amino acid region is conserved structurally at the sequence level, and this region also contains zinc-finger motifs associated with DNA binding [123]. This indicates that *disco* probably has retained its DNA binding and pupal and appendage patterning function in moths. We predicted the functional regions of *disco* in *B. mori* based on evolutionary conservation modelling and found that an additional 500 amino acids that were absent in *D. melanogaster* are predicted to be functional (conserved and exposed). Domain- and family-level modelling predicted at least two additional zinc-finger domains in this region (electronic supplementary material, figures S10 and S11). We also found many phosphorylated sites surrounding the zinc-finger domains. Examining mutations between *Anisota* and *Dryocampa disco* revealed three mutations mapped to these predicted functional regions (figure 5*b*). Given *disco’s* adult diel-specific expression in moths, its additional zinc-finger DNA binding domains and the high number of phosphorylated sites, we believe that it may be a candidate gene for diel-regulation in adult moths. we also identify other candidate genes for diel-niche evolution in Lepidoptera, which can be useful targets for further exploration in other diel-species pairs as well as functional validation using more direct experimental techniques.

## Data Availability

The transcriptome libraries are also archived on GenBank under BioProject PRJNA1102514, and all associated sequence data are archived on the SRA database (SRR28778930-SRR28778945). Datasets S1 to S17: Available at: 10.6084/m9.figshare.23661603. Supplementary material is available online [124].

## Declaration of AI use

ChatGPT 4 and Github Co-pilot were used to assist with creating the python code for data visualization for electronic supplementary material, figure S14.
